# Identification of castration-dependent and -independent driver genes and pathways in castration-resistant prostate cancer (CRPC)

**DOI:** 10.1186/s12894-022-01113-5

**Published:** 2022-10-18

**Authors:** Yan Li, Hui Shi, Zhenjun Zhao, Minghui Xu

**Affiliations:** 1 College of Life Sciences, Yantai University, 30th Qingquan Road, 264005 Yantai, Shandong Province China; 2 School of Life Sciences, Sun Yat-sen University, 510275 Guangzhou, Guangdong Province China

**Keywords:** Differentially expressed genes (DEGs), Castration-resistant prostate cancer (CRPC), Cell cycle, Cell proliferation, Prostate gland development

## Abstract

**Background:**

Prostate cancer (PCa) is one of the most diagnosed cancers in the world. PCa inevitably progresses to castration-resistant prostate cancer (CRPC) after androgen deprivation therapy treatment, and castration-resistant state means a shorter survival time than other causes. Here we aimed to define castration-dependent and -independent diver genes and molecular pathways in CRPC which are responsible for such lethal metastatic events.

**Methods:**

By employing digital gene expression (DGE) profiling, the alterations of the epididymal gene expression profile in the mature and bilateral castrated rat were explored. Then we detect and characterize the castration-dependent and -independent genes and pathways with two data set of CPRC-associated gene expression profiles publicly available on the NCBI.

**Results:**

We identified 1,632 up-regulated and 816 down-regulated genes in rat’s epididymis after bilateral castration. Differential expression analysis of CRPC samples compared with the primary PCa samples was also done. In contrast to castration, we identified 97 up-regulated genes and 128 down-regulated genes that changed in both GEO dataset and DGE profile, and 120 up-regulated genes and 136 down-regulated genes changed only in CRPC, considered as CRPC-specific genes independent of castration. CRPC-specific DEGs were mainly enriched in cell proliferation, while CRPC-castration genes were associated with prostate gland development. NUSAP1 and NCAPG were identified as key genes, which might be promising biomarkers of the diagnosis and prognosis of CRPC.

**Conclusion:**

Our study will provide insights into gene regulation of CRPC dependent or independent of castration and will improve understandings of CRPC development and progression.

**Supplementary Information:**

The online version contains supplementary material available at 10.1186/s12894-022-01113-5.

## Background

One of the most common malignancies in man is prostate cancer (PCa), which also causes the second highest deaths in man. Absolutely, PCa arises as an androgen driven disease [1]. Therefore androgen deprivation therapy (ADT) was adopted as the major treatment for metastatic hormone-sensitive PCa, by suppressing circulating testosterone to decrease its level to “castrate levels” ( < = 50 ng/dL) [2] and induce of apoptosis [3]. However, although the initial treatment effect is significant, almost all PCa patients ultimately progress and castration resistant prostate cancer (CRPC) ensue, which is associated with high mortality rates [4]. CRPC represents a particular stage in the continuum of the disease and is defined by continuous rise of prostate specific antigen levels in serum or/and progression of metastatic spread in the setting of castrate levels of testosterone [5–7].

Nowadays, suppression of androgen receptor (AR) signaling through ADT has remained the first-line treatment for men with advanced PCa. ADT includes surgical or medical castration by using luteinizing hormone-releasing hormone (LHRH) agonists or antagonists with or without anti-androgen drugs [8]. Exposure to LHRH agonists downregulates the LHRH receptor, decreasing LH release and inhibiting testosterone production, while LHRH antagonists, such as cetrorelix and abarelix, directly inhibit the LHRH receptor, resulting in the decreased production of LH and testosterone. Surgical bilaterial castration also decreases testosterone levels by removing the testes, the source of its production organ, although surgical castration has been practically set aside for some time, given the spread of ADT [9]. However, by using LHRH agonists or antagonists, for nearly all patients, after 1–2 years remission, cancer cells become resistant with the emergence of metastatic CRPC [10]. Today’s standard of care for advanced PCa includes gonadotropin-releasing hormone agonists (leuprolide), second-generation nonsteroidal AR antagonists (apalutamide, enzalutamide and darolutamide) and the androgen biosynthesis inhibitor abiraterone [11]. Currently, with minimal additional toxicity, abiraterone acetate has been proved to be a well-tolerated, convenient and effective treatment option [12].

Besides the above mentioned drugs for the treatment of CRPC, AR-V7, the androgen receptor isoform encoded by splice variant 7, has become an attractive biomarker predicting the effect of androgen signaling inhibitor [10, 13]. AR-V7 lacks the ligand binding domain and is the target of enzalutamide and abiraterone. It is supposed that detection of AR-V7 in circulating tumor cells from patients with CPRC might be closely related to resistance to enzalutamide and abiraterone [14].

Many mechanisms of CRPC have been proposed, including reactivation of androgen synthesis, up-regulation of genes related to androgen metabolism, and reprogramming of the tumor microenvironment [13]. However, CRPC treatment is challenging [15]. Thus, identifying effective molecular biomarkers and therapeutic targets of CRPC is of ultra-importance for PCa prediction and treatment. Previously, Microarray techniques have been used to identify biomarkers and targets in CRPC. For example, D’Antonio et al. [16] identified pathways of androgen independence of CRPC by comparing PCa cells (androgen-independent) with LNCaP cells (androgen-dependent). Terada et al. [17] found that prostaglandin E2 receptor subtype 4 (EP4) is a central gene in CRPC by comparing a mouse xenograft model of PCa with later-derived CRPC.

The biological functions of the mammalian epididymis, an important male accessory gland, are substantially affected by androgen [18]. The mammalian epididymis is an important male accessory gland with many biological functions, including maturation and concentration of spermatozoa produced by testis, secretion and resorption of different molecules and proteins, and storage of spermatozoa. The development and normal functions of the epididymis are regulated mainly by androgens and testicular factors [19]. Bilateral castration led to systematic changes of epididymal gene expression profile [20]. It would be useful to identify critical androgen-related pathways in the epididymis to help understand CRPC and identify biomarkers or targets, thus providing opportunities to combat the disease.

**Fig. 1:**
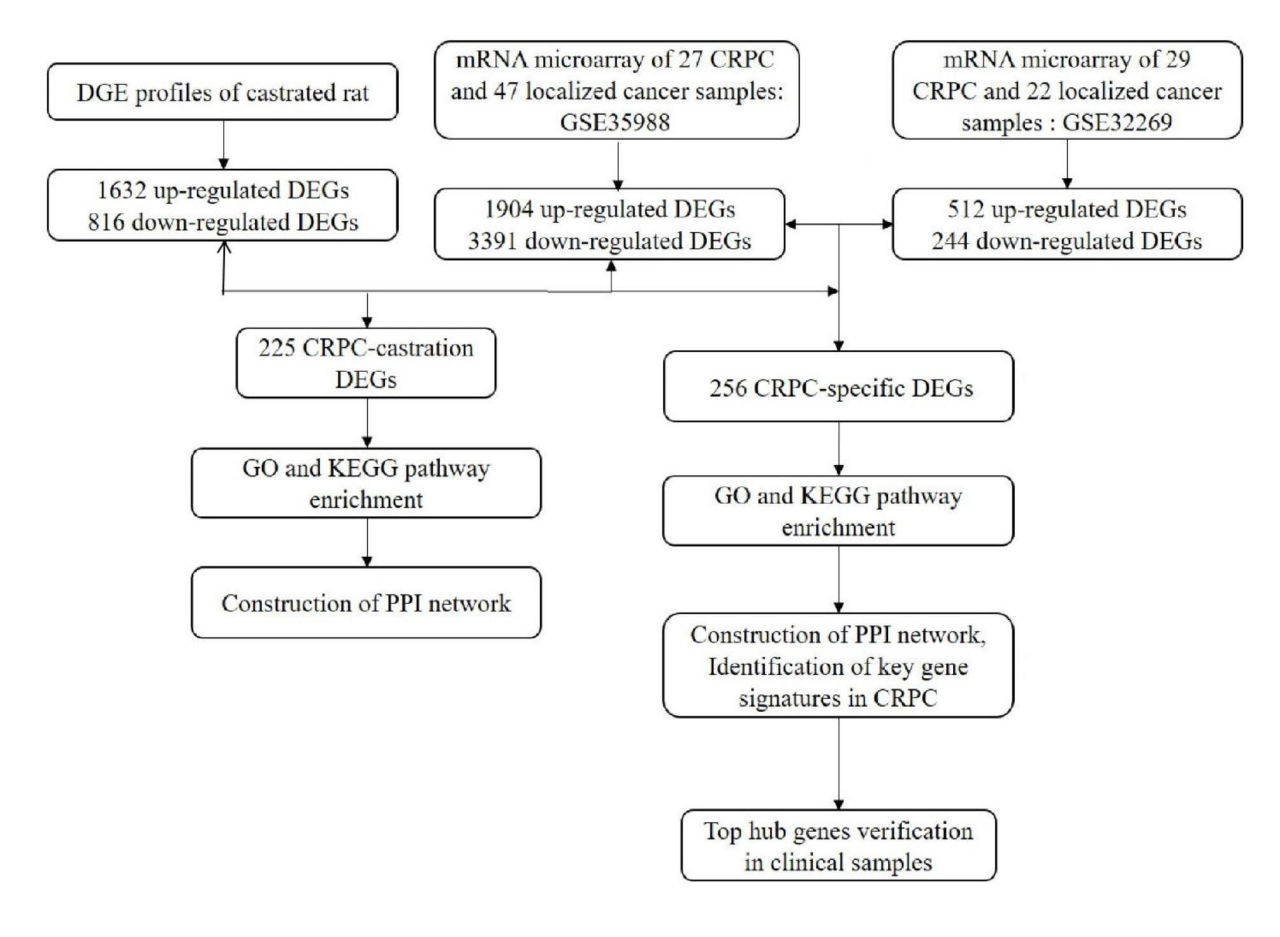
Flow diagram showing an overview of the study

Compared with microarray, digital gene expression profiling provides absolute gene expression measurements and an unbiased view of the whole transcriptome with greater precision and sensitivity. Here we identified differentially expressed genes in the epididymides of sham-operated control and bilateral castrated male mature rats on the 7th day after surgery by using the DGE system on the whole-genome scale, as previous studies have demonstrated that gene expression profile is most likely to reflects transcriptional changes in surviving epididymal cells on the 7th day of post castration because apoptotic cell death is no longer detected at this moment [20]. Then we combined our data with two published datasets to identify differentially expressed genes (DEGs) between CRPC and primary PCa. We obtained DEGs of either dependent (CRPC-castration) or independent (CRPC-specific) of castration to investigate the role of castration in CRPC. Functional enrichment analysis, protein-protein interaction (PPI) network, and survival analysis of these DEGs showed distinct functional roles of CRPC-castration and CRPC-specific genes (Fig. 1). CRPC-specific DEGs were mainly enriched in cell proliferation, while CRPC-castration genes were associated with prostate gland development. In summary, our study provided insights into a deeper understanding of CRPC pathogenesis.

## Methods

### Microarray data and DGE profiling

Two gene expression profiles (GSE35988, GSE32269) were obtained from Gene Expression Omnibus (GEO, https://www.ncbi.nlm.nih.gov/geo). GSE35988 contained 27 metastatic CPRC samples and 47 localized PCa samples, and GSE32269 was composed of 29 metastatic CPRC samples and 22 primary PCa samples.

### Animals and castration procedure

Twenty healthy and mature male Sprague-Dawley (SD) rats (12 weeks old and the body weight reached 350 ~ 400 g) were obtained from the Animal Research Centre of the Lv Ye Pharmaceutical Company (Yantai, China) and were housed at an environmental temperature of 20 ± 2 °C, receiving 12 h of light and 12 h of dark, with food and water provided ad libitum. Rats were randomly divided into two groups by using a random number Table (10 animals of each group), namely sham-operated control group (Con-EP) and castrated group (Cas-EP). Before castration treatment, rats were anesthetized by intraperitoneally injection of sodium pentobarbital (30 mg/kg body weight). Thereafter, the efferent ducts and the testicular arteries and veins were ligated, then the testes were removed from the ligation point, leaving the intact epididymides in the scrotal area. In the meantime, the sham operation was also taken for the control group. Analgesia was provided by administration of meloxicam Q 24 h (1 mg/kg PO or SC) (Metacam, Boehringer Ingelheim, St Joseph, MO) the day prior, the day of and the day following surgery. No obvious behavioral changes were observed in both control and the operation group. At the conclusion of the experiment, all the surviving rats were intraperitoneally anesthetized with sodium pentobarbital (30 mg/kg body weight) and their necks were dislocated for euthanasia. The whole epididymides were taken out from the castrated and sham-operated control rats after 7 days’ recovery and were immediately frozen in liquid nitrogen and stored at -80 ℃ before use. Protocols for the use of animals in these experiments were approved by the Research Animal Care and Use Committee of Yantai University (the approval reference number: YD.No20190916S0350210[315]) and were carried out in strict accordance with Guidelines for Ethical Review of Laboratory Animal Welfare of China. Ethical approval was obtained before the initiation of the research work and all efforts were made to minimize suffering.

### Library preparation and sequencing

Total RNA was isolated from the epididymides from the control group and castrated group, respectively, by using RNAiso Plus reagent (TaKaRa, Dalian, China). The purity and quality of total RNA were evaluated by Agilent 2000 and were considered sufficient for subsequent library construction and sequencing. Tag libraries for the samples were prepared using the Illumina gene expression sample, Prep Kit. Each sample of 6 µg of the total RNA was extracted and purified by oligo (dT) magnetic bead adsorption. Primed by oligo (dT), mRNA bound to the bead was used as the template to synthesize a double-stranded cDNA. The 5’ ends of tags were digested with restriction enzyme *Nla*III at CATG sites. The cDNA fragments with 3’ ends connected to Oligo(dT) beads were purified and the Illumina adaptor 1 was ligated to the 5’ ends. The junction of Illumina adaptor 1 and CATG site is the recognition site of MmeI, which is a type of endonuclease with separated recognition sites and digestion sites. It cuts at 17 bp downstream of the CATG site, producing tags with adaptor 1. After removing 3’ fragments with magnetic beads precipitation, Illumina adaptor 2 is ligated to the 3’ ends of tags, acquiring tags with different adaptors of both ends to form a tag library. After 15 cycles of linear PCR amplification, 105 bp fragments were purified by 6% TBE PAGE Gel electrophoresis. After denaturation, the single-chain molecules were fixed onto the Illumina Sequencing Chip (flowcell). Each molecule was grown into a single-molecule cluster sequencing template through Situ amplification. Then, they were sequenced with the method of sequencing by synthesis (SBS). Each tunnel generated millions of raw reads with a sequencing length of 49 bp.

### Tag annotation and gene expression level quantification

Five steps were needed to transform raw tags into clean tags: (1) removal of the 3’ adaptor to preserve the 21 nt long sequences; (2) removal of the empty reads (reads with only adaptor sequences but no tags); (3) removal of the low-quality tags (tags with unknown sequences); (4) removal of too long or too short tags, keeping the 21 nt tags; (5) removal of tags with only one copy number (probably because of sequencing error). To convert digital profiles to gene expression, the tag sequences were mapped to the reference genes of rat from NCBI, with no more than 1 nucleotide mismatch. Clean tags mapped to multiple loci were filtered, only unambiguous mapped tags were kept. To identify gene expression, the number of unambiguous tags for each gene was calculated and then normalized to the number of transcripts per million tags (TPM). Gene ontology was assigned to all mapped genes to annotate their possible functions.

### Real-time quantitative PCR analysis of genes

Real-time quantitative PCR (qPCR) was performed to examine the relative gene expression levels. Briefly, by using ReverTra Ace reverse transcriptase (Toyobo Co., Osaka, Japan), 1 ng ~ 1 µg total RNA was reverse-transcribed into cDNA with oligo(dT)_18_, and then qPCR was performed on the QIAGEN’s Roter-Gene Q instrument by using Platinum SYBR Green qPCR SuperMix-UDG (Life Technologies, Cat. no.: 11733-038, CA, USA). In each reaction, 20 µL reaction mixtures containing 1 µL cDNA were incubated at 95℃ for 5 min, followed by 40 cycles of 95℃ for 10 s and 60℃ for 45 s. GAPDH was taken as an endogenous reference. 2^−△△Ct^ method was used to calculate the differences of the expression level of genes in samples examined [21]. All experiments were run in triplicate and results were shown as the mean ± SD (n = 10). The primer sequences were available in Table 1.

**Table 1:** Primer sequences of qPCR

Gene	Primer sequence (5’-3’)
*Nfkbie*	F: GTGGACTGGATGGAGATTCTTG R: TTTCCTGGTGGCTGGTAATG
*Plscr4*	F: CAGCTTGGGACACTAGGTTATT R: GGGAACTAAGGGCGTCATTT
*Apoc1*	F: ACAAGGACAGGGTAGAGAAGA R: ACAGGAAGTGCGATGAAGAG
*Lcn8*	F: GGGTAGAAGGCTTGTTCCTTAC R: CTCTTTCTGAACCCACTGATCTT
*Prdx1*	F: TGTGTCCCACGGAGATCATTGCTT R: TGTTCATGGGTCCCAATCCTCCTT
*Prdx3*	F: TGGTGTCATCAAGCACCTGAGTGT R: AAGCTGTTGGACTTGGCTTGATCG
*GAPDH*	F: TGGGTGTGAACCACGAGAA R: GGCATGGACTGTGGTCATGA

### Identification of DEGs of digital profiling data

To identify the differentially expressed genes in the epididymis of rats after bilateral castration, a modified algorithm according to Audic S, et al. was used [22]. Suppose the number of clean tags corresponding to gene A was x, the expression level of each gene was only a small fraction of all genes expression level, therefore, p(x) follows the Poisson distribution. Furthermore, suppose the number of clean tags of sample 1 and 2 was N_1_ and N_2_, respectively, while the clean tags of gene A in sample 1 and 2 were x and y, respectively. The probability of gene A expression was equal in two samples could be calculated as the formula:

*P*(*y*|*x*)=($$\frac{{N}_{2}}{{N}_{1}}$$)^*y*^$$\frac{\left(x+y\right)!}{{x!y!(1+{N}_{2}/{N}_{1})}^{(x+y+1)}}$$

The acquired p-value was controlled with false discovery rate (FDR) using Benjamini-Hochberg method [23]. Differentially expressed genes with FDR ≤ 0.001 and log_2_ fold-change ≥ 1 were extracted. The correlation between the two libraries was measured by the Pearson correlation coefficient.

### Identification of DEGs of microarray data

Online analysis tool GEO2R (https://www.ncbi.nlm.nih.gov/geo/geo2r) and R software (v4.0.5; https://www.r-project.org) were used for the identification of DEGs. The raw data from GSE35988 on GPL6480 and GSE32269 on GPL96 were normalized, transformed into expression values with GEO2R. Then empirical Bayes methods were applied to identify DEGs between CRPC and primary PCa with the limma package [24]. DEGs were defined with false discovery rate (FDR) < 0.001 and |log2(fold change)|>1 [25].

### Functional enrichment and PPI network analysis of DEGs

The Database for Annotation, Visualization, and Integrated Discovery (DAVID; http://www.david.niaid.nih.gov) [26] and the R package clusterProfiler were used to perform GO and KEGG pathway analyses separately on CRPC-castration and CRPC-specific DEGs. Search Tool for the Retrieval of Interacting Genes/ Proteins (STRING; https://string-db.org) was used to construct PPI networks [27, 28]. Protein interactions with combined scores of > 0.15 were selected for the PPI network construction. Further, the Cytoscape software (v3.8.2) was utilized to calculate the node degree by MCODE and CytoHubba apps [29].

## Results

### Dramatic transcriptional changes in rats’ epididymis after bilateral castration

To explore the transcriptional differences in the epididymis between Con-EP and Cas-EP rats, we obtained more than 4 million clean tags for each library. As demonstrated in Additional file 1, the distribution of total clean tags and distinct clean tags showed similar patterns in both DGE libraries, showing no bias between the library construction of Con-EP and Cas-EP. Around 3 million tags were mapped to genes for both Con-EP and Cas-EP, accounting for 62.89% and 70.32%, respectively.

**Fig. 2:**
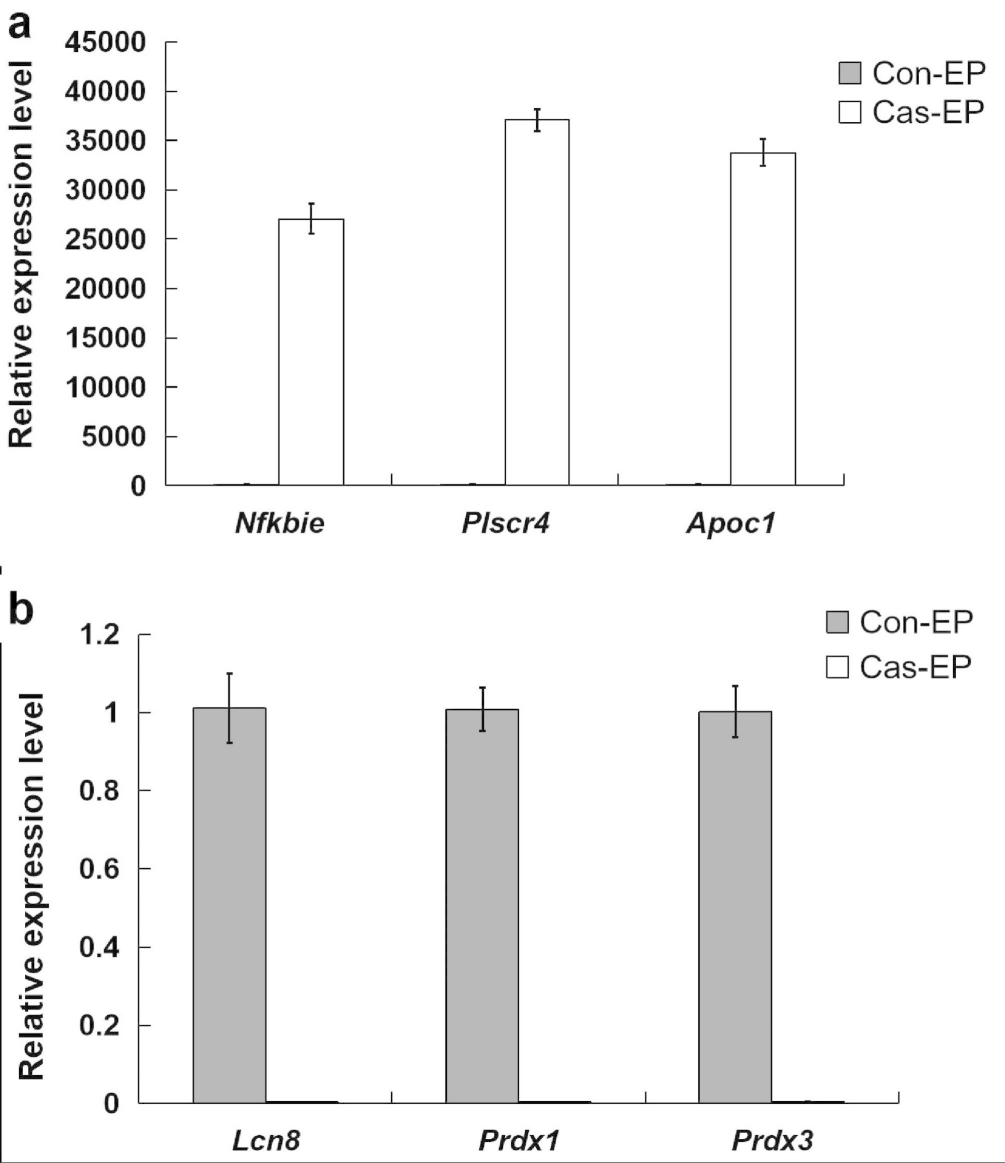
Real-time fluorescent quantitative PCR analysis of differentially expressed genes before and after castration. a: down-regulated genes after bilateral castration, b: genes up-regulated after bilateral castration

A total of 2,448 genes changed significantly between the two libraries. Among these genes, 1,632 were up-regulated while 816 were down-regulated after bilateral castration. More than 90% of the genes (2,274) were up- or down-regulated between 1.0 and 5.0 fold (data not shown). The Pearson correlation coefficient of the two libraries was only 0.658, reflecting the tremendous influence of castration on the gene expression profile of rat epididymis. We then performed qPCR of six selective genes which were up-regulated (*Nfkbie*, *Plscr4* and *Apoc1*) or down-regulated (*Lcn8*, *Prdx1* and *Prdx3*) to confirm the validity of the differentially expressed genes identified by DGE (n = 10). As expected, we obtained results that coincided with our sequencing data, confirming the authenticity of our sequencing results (Fig. 2).

### CRPC-castration genes were enriched in cell proliferation and prostate gland development

**Fig. 3:**
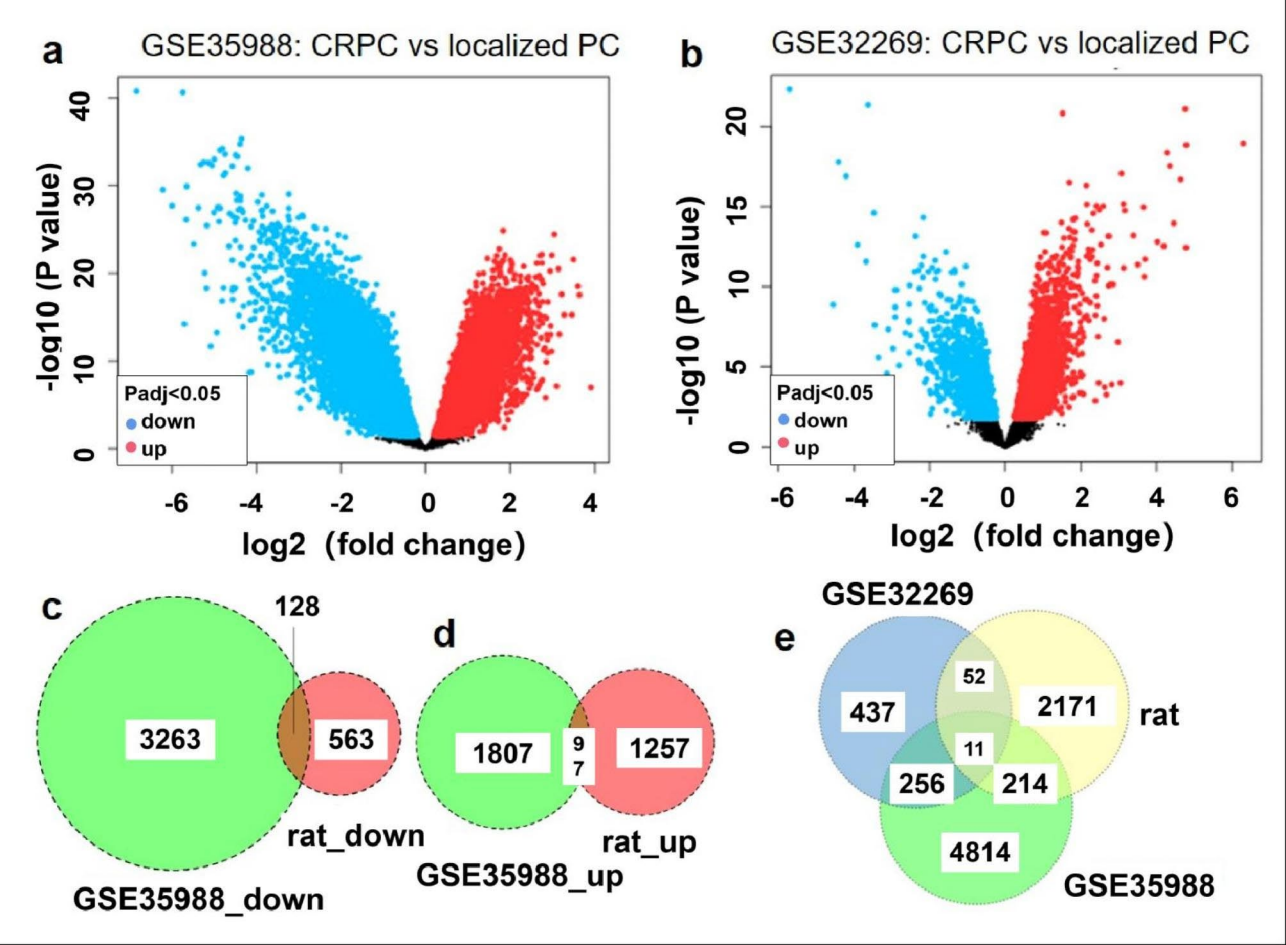
Database analysis. a and b: Volcano plots of all genes in GSE35988 and GSE32269 Red dots represent genes with fold change ≥ 2 and P < 0.001, blue dots represent genes with fold change ≤ − 2 and P < 0.001, and the other dots represent the rest of genes with no statistically significant change in expression. FC, fold change. c, d and e: Venn diagrams showing the number of genes expressed as common and unique between the identified DEGs in DGE profiles of castrated rat and genes in the NCBI-gene database

To obtain potential CRPC driver genes dependent or independent of castration, we identified DEGs in the CRPC samples compared with the primary PCa samples. We obtained 1,904 up-regulated genes and 3,391 down-regulated genes (fold change > 1, P < 0.001) from GSE35988 (Fig. 3a), and 512 increased genes and 244 decreased genes (fold change > 1, P < 0.001) from GSE32269 (Fig. 3b). A total of 97 up-regulated genes (Fig. 3d) and 128 down-regulated genes (Fig. 3c) were shared by CRPC (GSE35988) and testis castration (data not shown), considered as CRPC DEGs related to castration (CRPC-castration). In contrast to testis castration, 120 up-regulated genes and 136 down-regulated genes changed only in CRPC (both GSE35988 and GSE32269) (Fig. 3e, **data not shown**), considered as CRPC-specific DEGs independent of castration.

**Fig. 4:**
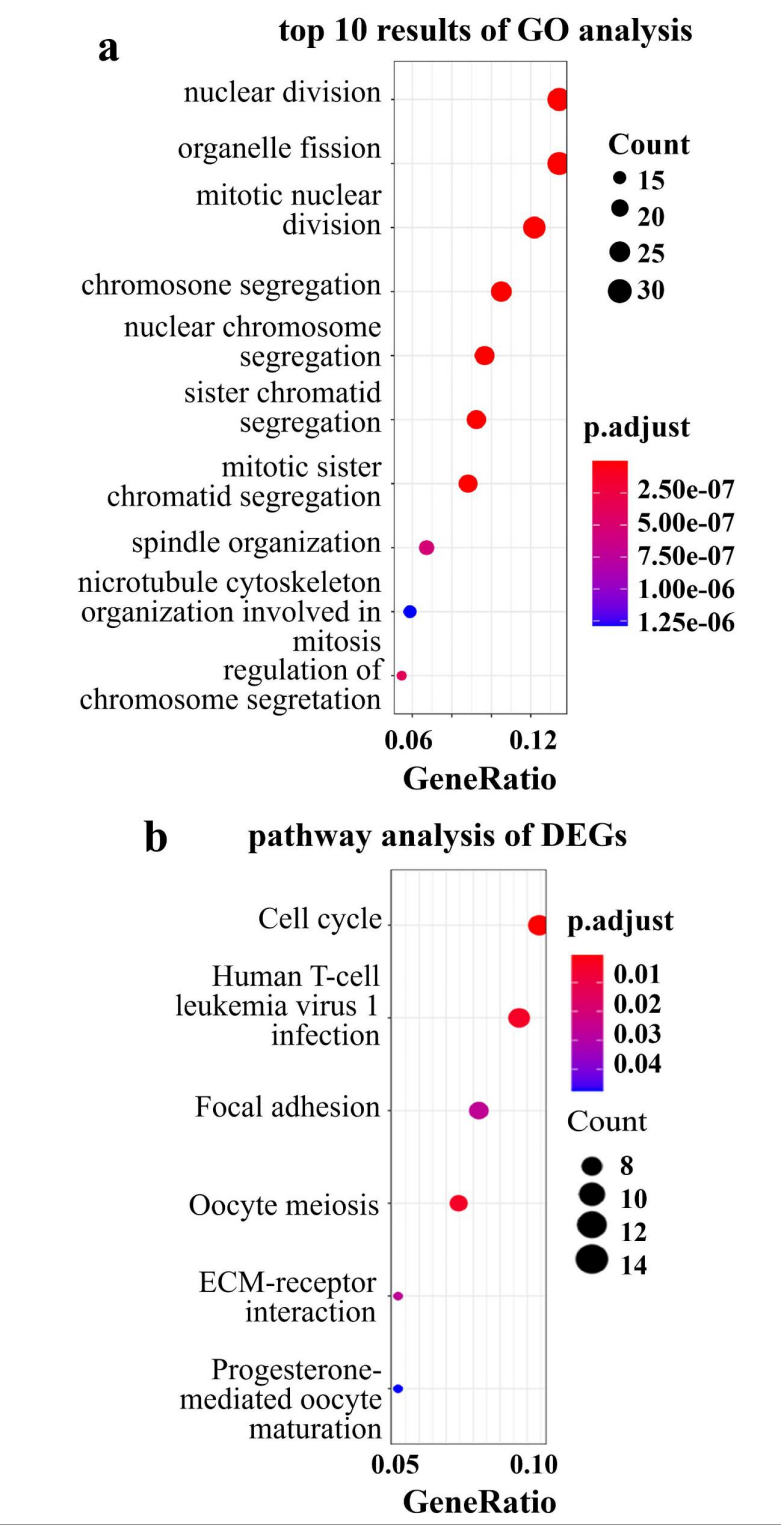
Go and KEGG analysis. a: The top 10 results of GO functional analysis in CRPC-specific DEGs. b: KEGG functional analysis in CRPC-specific DEGs.

**Fig. 5:**
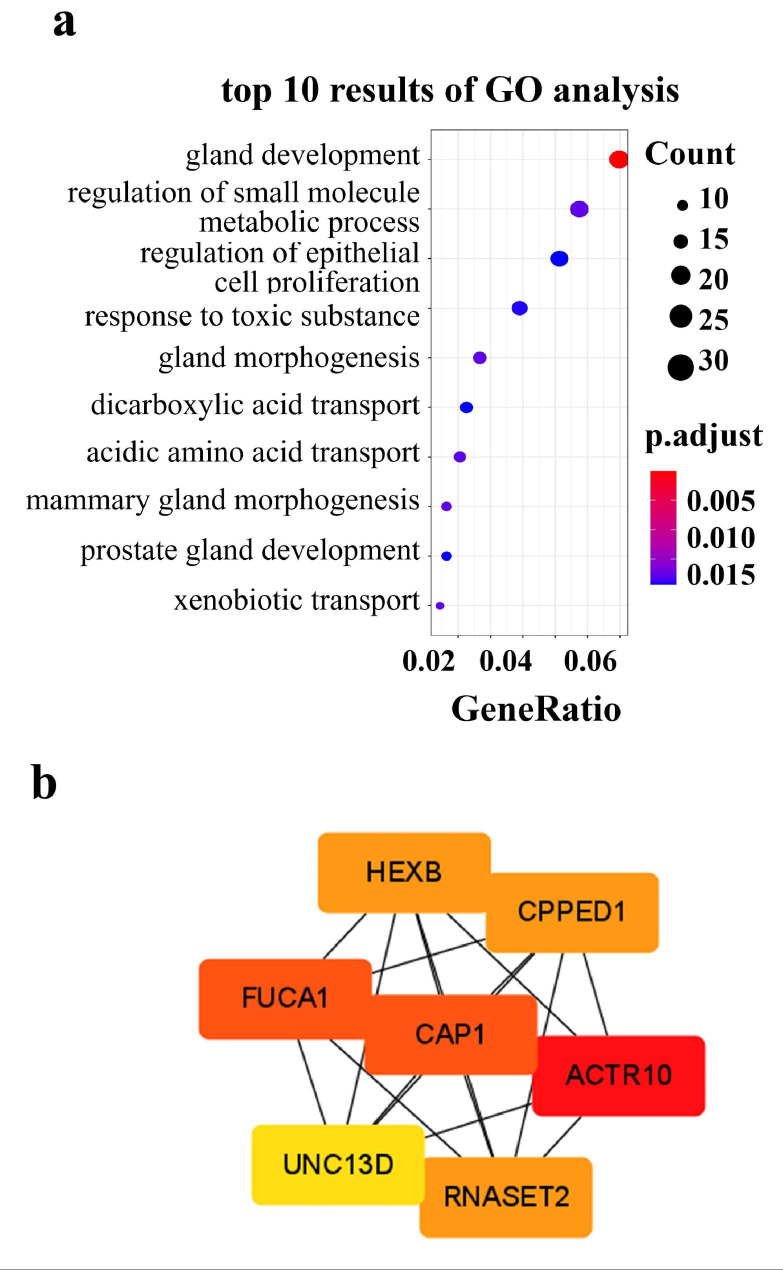
Go and PPI analysis. a: The top 10 results of GO functional analysis in CRPC-castration DEGs. b: Construction of PPI network of CRPC-castration DEGs.

Next, we explored the functional enrichment of these two lists of DEGs by using GO and KEGG analysis. The CRPC-specific DEGs significantly enriched cell division or mitotic processes (Fig. 4a), consistent with their oncogenic role in tumor cell proliferation. Concordantly, pathway analysis identified enrichment in the cell cycle pathway and focal adhesion pathway (Fig. 4b) (**Additional file 2**). On the other hand, GO enrichment of CRPC-castration DEGs (data not shown) showed that gland development, epithelial cell proliferation, and prostate gland development were substantially affected by castration (Fig. 5a). Pathway analysis showed that the pyruvate metabolism pathway was significantly enriched (**Additional file 3**).

**Fig. 6:**
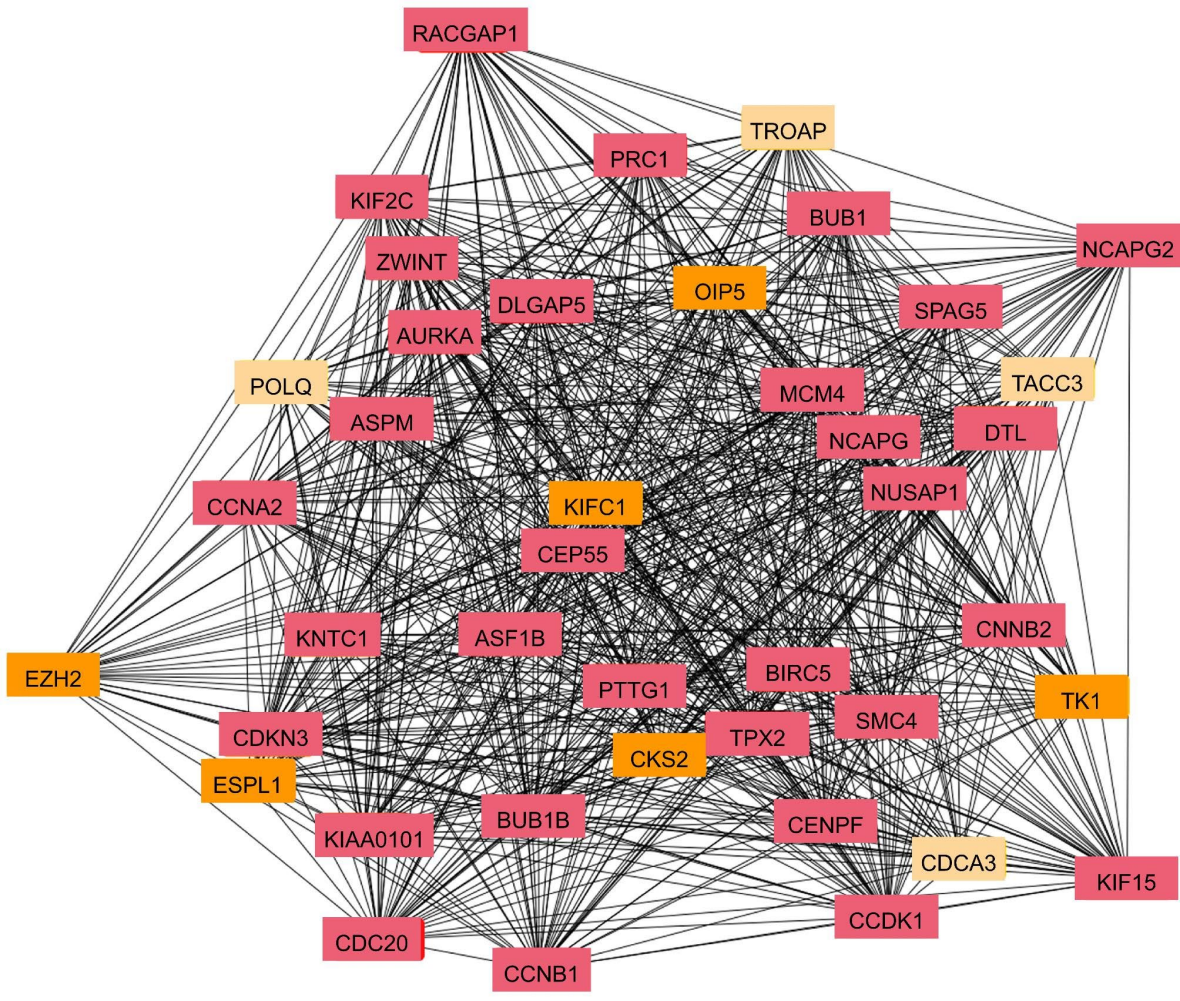
Top 41 of PPI network of CRPC-specific DEGs.

Furthermore, we used STRING to explore the protein-protein interaction (PPI) of the DEGs. CRPC-specific DEGs formed a PPI network with 208 nodes and 1353 edges. Among the 41 hub genes (node degree > 30) were chromatin remodeling factors EZH2 and PRC1, and cell cycle regulators CDCA3, CCNA2, CCNB2, CDKN3, CDK1, CCNB1, CDC20, MCM4, SMC4, CENPF, BUB1, BUB1B, NUSAP1, and NCAPG (Fig. 6). In contrast, CRPC-castration DEGs formed PPI networks with 171 nodes and sparse connections. The most connected sub-network with the hub gene ACTR10 was involved in innate immunity and cell polarity (Fig. 5b).

**Fig. 7:**
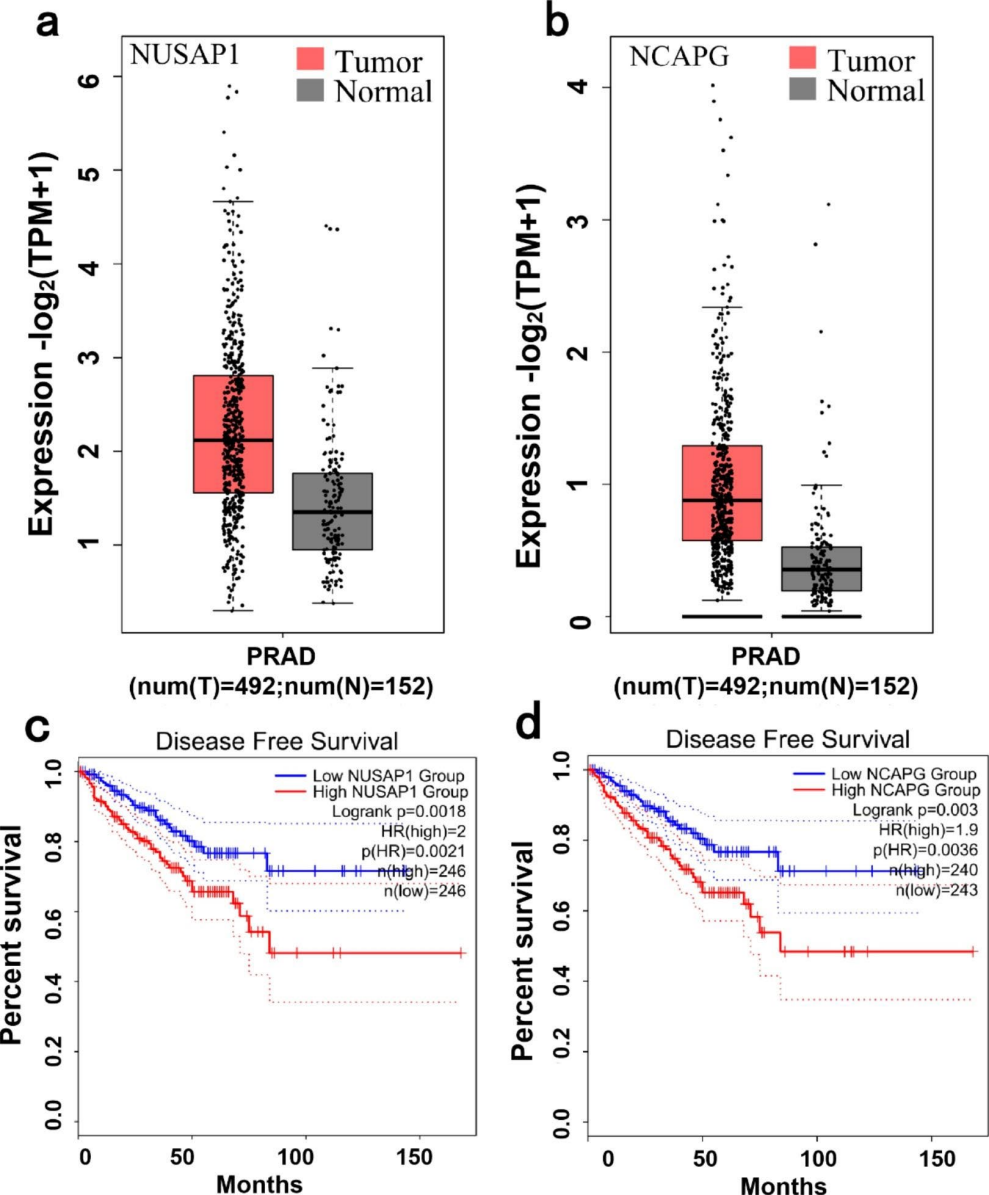
Key genes analysis. a and b: Expression of the key genes NUSAP1 and NCAPG in TCGA with GEPIA. c and d: Disease-free survival in relation to the expression of the key genes (NUSAP1 and NCAPG) in TCGA.

Finally, based on network and pathway analyses, we identified two key genes (NUSAP1 and NCAPG) among the top 41 nodes in the CRPC-specific PPI network, which participate in various pathways including chromosome segregation and nuclear division. Further analysis showed that, for both genes, higher expression was associated with shorter disease-free survival of patients (Fig. 7c and d). Interestingly, the expression of NUSAP1 and NCAPG differed between primary PCa and normal prostate tissues (Fig. 7a and b), which led us to hypothesize that they may have a role both in the initiation and in the progression of PCa.

## Discussion

In this research, we focused on the role of bilateral castration-related genes in tumors and genes that are associated with tumors after blocking out these bilateral castration-related genes. We performed in silico analysis with DEG data in this study and publicly available CPRC-associated gene expression profiles datasets, and identified two significant genes, namely NUSAP1 and NCAPG in CRPC. NUSAP1 and NCAPG participated in various pathways including chromosome segregation, sister chromatid segregation, nuclear division. In consideration of the important role of these pathways in the initiation, progression and metastasis in PCa, it’s reasonable to assume that NUSAP1 and NCAPG play a critical role in the initiation and progression of PCa. In accordance with our hypothesis, a number of authoritative papers have reported that NUSAP1 plays a key role in cancers including PCa [30, 31]. Current understanding of NUSAP1’s function in specific mechanisms of cancer is limited, although some data indicates it could be a potential marker of cell proliferation [32]. On the molecular and cellular level, NUSAP is an essential microtubule-stabilizing and bundling protein that crosslinks microtubules at the central part of the spindle during mitosis [33]. Li’s group further reveals that NUSAP functions as a modulator of the dynamics of kinetochore microtubules and plays a pivotal role in chromosome oscillation [34]. In PCa cell lines, NUSAP1 is proved to be regulated by retinoblastoma-associated protein 1 (RB1), whose knockdown upregulated the expression of NUSAP1 via the RB1/E2F1 axis [35]. Furthermore, elevated expression level of NUSAP1 may increase proliferation and invasion of PCa cells, positively affecting prostate cancer progression. Despite limited effects on cell proliferation, NUSAP1 participates in development of metastatic disease, possibly by regulation of the expression of family with sequence similarity 101 member B (FAM101B). FAM101B is associated with the organization of perinuclear actin networks as well as the regulation of nuclear shape during epithelial-mesenchymal transition (EMT), a crucial event involved in the invasion and spread of cancer cells [36]. Besides, FAM101B functions as a signaling effector of TGF-β1, which has been widely demonstrated to promote invasion and metastatic spread during the progression of prostate tumor [37]. In other types of cancer, gastric cancer for example, low NUSAP1 expression is proven to inhibit mTORC1 pathway, hence suppressing proliferation, migration, and invasion of cancer cells [38].

NCAPG has not been well studied in CRPC. NCAPG is a subunit of condensing | complex and is involved in proper segregation of sister chromatids during the cell cycle. There are several miRNA published involved in CRPC. Current research showed that NCAPG is targeted by miR-145-3p, a passenger strand downregulated in CRPC. High expression of NCAPG in CRPC compared with hormone-sensitive prostate cancer suggested that NCAPG is closely associated with the pathogenesis of CRPC [39]. Another CRPC-related passenger strand miR-99a-3p was also found to downregulate NCAPG significantly, indicating potent antitumor effects [40]. A recent study showed that overexpression of NCAPG promoted cell proliferation and decreased cell apoptosis in hepatocellular carcinoma via activating PI3K-AKT signaling pathway [41]. Further studies about the concrete mechanism of NCAPG regulation in CRPC are in progress.

## Conclusion

Our study provided an insight into the regulation of epididymal gene expression after bilateral castration. These results contribute to our understanding of testis-dependent epididymal functions. Still, we preformed insights into gene regulation of CRPC dependent or independent of castration and improved our understanding of CRPC development and progression.

## Electronic supplementary material

Below is the link to the electronic supplementary material.

Supplementary Material 1

## Data Availability

The datasets used and/or analysed during the current study available from the corresponding author on reasonable request.
