# Supplementary material for: Identification of castration-dependent and -independent driver genes and pathways in castration-resistant prostate cancer (CRPC)
Source: BMC Urol. 2022 Oct 18;22:162. doi: 10.1186/s12894-022-01113-5 (PMC9580185; doi:10.1186/s12894-022-01113-5)
Supplement: Supplementary file 1 — Supplementary Material 1 [file 12894_2022_1113_MOESM1_ESM.docx]

**Additional files**

**Additional file 1:** Distribution of copy number of the clean tags.

**Additional file 2:** KEGG pathway enrichment analysis of CRPC-specific DEGs.

**Additional file 3:** KEGG pathway enrichment analysis of CRPC-castration DEGs.

**Additional file 1**

|  | **Con-EP Total Clean Tags** | **Con-EP Distinct Clean Tags** | **Cas-EP Total Clean Tags** | **Cas-EP Distinct Clean Tags** | | **Con-EP Matched clean Tags / Clean Tags** | **Cas-EP Matched clean Tags / Clean Tags** |  |
| --- | --- | --- | --- | --- | --- | --- | --- | --- |
| [2, 5] | 160340, 3.44% | 57179, 58.48% | 188648, 4.21% | 67597, 60.56% | 2930926/4660635, 62.89% | | 3152446/4483123, 70.32% |  |
| [6, 10] | 101916, 2.19% | 13436, 13.74% | 112910, 2.52% | 14931, 13.38% |  |  |  |  |
| [11, 20] | 134558, 2.89% | 9214, 9.42% | 144901, 3.23% | 9897, 8.87% |  |  |  |  |
| [21, 50] | 257009, 5.51% | 7963, 8.14% | 274060, 6.11% | 8546, 7.66% |  |  |  |  |
| [51, 100] | 288662, 6.19% | 4055, 4.15% | 295970, 6.60% | 4174, 3.74% |  |  |  |  |
| >100 | 3718190, 79.78% | 5931, 6.07% | 3466634, 77.33% | 6482, 5.81% |  |  |  |  |

**Additional file 2**

| **ID** | **Description** | **GeneRatio** | **BgRatio** | **pvalue** | **p.adjust** | **qvalue** | **Count** |
| --- | --- | --- | --- | --- | --- | --- | --- |
| hsa04110 | Cell cycle | 14/134 | 124/8111 | 1.32E-08 | 2.94E-06 | 2.74E-06 | 14 |
| hsa04114 | Oocyte meiosis | 10/134 | 129/8111 | 5.02E-05 | 0.004638961 | 0.004333217 | 10 |
| hsa05166 | Human T-cell leukemia virus 1 infection | 13/134 | 219/8111 | 6.27E-05 | 0.004638961 | 0.004333217 | 13 |
| hsa04510 | Focal adhesion | 11/134 | 201/8111 | 0.000472739 | 0.026237016 | 0.024507786 | 11 |
| hsa04512 | ECM-receptor interaction | 7/134 | 88/8111 | 0.000599267 | 0.026607472 | 0.024853826 | 7 |
| hsa04914 | Progesterone-mediated oocyte maturation | 7/134 | 100/8111 | 0.001282954 | 0.04746928 | 0.044340674 | 7 |

**Additional file 3**

| **ID** | **Description** | **GeneRatio** | **BgRatio** | **pvalue** | **p.adjust** | **qvalue** | **Count** |
| --- | --- | --- | --- | --- | --- | --- | --- |
| hsa00620 | Pyruvate metabolism | 6/120 | 47/8111 | 6.05E-05 | 0.016098971 | 0.014525388 | 6 |
